# Exostosin1 as a novel prognostic and predictive biomarker for squamous cell lung carcinoma: A study based on bioinformatics analysis

**DOI:** 10.1002/cam4.3643

**Published:** 2020-12-13

**Authors:** Disheng Wu, Chao Huo, Siyu Jiang, Yanxia Huang, Xuehong Fang, Jun Liu, Min Yang, Jianwei Ren, Bilian Xu, Yi Liu

**Affiliations:** ^1^ Guangdong Key Laboratory for Research and Development of Natural Drugs, Department of Pharmacology Marine Medicine Research Institute, Guangdong Medical University Zhanjiang Guangdong China; ^2^ Department of Anus and Intestines Shenzhen Nanshan District People's Hospital Shenzhen Guangdong China; ^3^ Department of Pharmacy The Third People‘s Hospital of Shantou Shantou Guangdong China; ^4^ Shenzhen Ritzcon Biological Technology Co., Ltd. Shenzhen Guangdong China; ^5^ Department of Pharmacology Guangdong Medical University Zhanjiang Guangdong China

**Keywords:** bioinformatics analysis, biomarker, *EXT1*, lung squamous cell carcinoma, prognosis

## Abstract

The exostosin (EXT) protein family is involved in diverse human diseases. However, the expression and prognostic value of EXT genes in human lung squamous cell carcinoma (LUSC) is not well understood. In this study, we analyzed the association between expression of *EXT1* and *EXT2* genes and survival in patients with LUSC using bioinformatics resources such as Oncomine and The Cancer Genome Atlas (TCGA) databases, the Gene Expression Profiling Interactive Analysis (GEPIA) server and Kaplan–Meier plotter. Furthermore, regulatory microRNAs (miRNAs) were predicted for *EXT1* and used to establish a potential miRNA‐messenger RNA (mRNA) regulation network for LUSC using the ENCORI platform. We observed that *EXT1* and *EXT2* expression levels were higher in LUSC than those in normal tissues. However, only *EXT1* expression was significantly associated with poor overall survival (OS) in LUSC patients. Functional annotation enrichment analysis showed that genes co‐expressed with the *EXT1* gene were enriched in biological processes such as cell adhesion and migration, and KEGG pathways such as extracellular matrix receptor interactions, complement and coagulation cascades, and cell death. Furthermore, three miRNAs, hsa‐mir‐190a‐5p, hsa‐mir‐195‐5p, and hsa‐mir‐490‐3p, were identified to be potentially involved in the regulation of *EXT1*. In summary, we identified *EXT1* expression as a novel potential prognostic marker for human LUSC and the regulatory miRNAs that could possibly contribute to the prognosis of the disease.

## Graphical abstract

*EXT1* expression as a novel potential prognostic marker for human lung squamous cell carcinoma and the regulatory miRNAs that could possibly contribute to the prognosis of the disease.

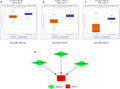

## 1 INTRODUCTION

Heparin sulfate proteoglycans (HSPGs) are ubiquitous components of the extracellular matrix and play an important role in tissue homeostasis.^1^ Extensive research has demonstrated that heparin sulfate (HS) is essential for signal transduction in various processes such as cell survival, division, migration, differentiation, and cancer development.^2^ The exostosin (EXT) family of glycosyltransferases, including *EXT1* and *EXT2*, mediate the synthesis of HS.^3^ Both genes that encode exostosin glycosyltransferases (*EXT1* and *EXT2)* function as tumor‐suppressors,^4^ although the molecular mechanisms and prognostic value of exostosins (EXTs) in cancer is still unclear.

The *EXT1* gene, located on chromosome 8, encodes an endoplasmic reticulum‐resident type II transmembrane glycosyltransferase involved in the chain elongation step of HS biosynthesis. Mutations in this gene cause the type I form of multiple exostoses. Furthermore, *EXT1* is overexpressed in various cancers such as adult acute lymphoblastic leukemia (ALL),^5^ hepatocellular carcinoma (HCC)^6^ and breast cancer.^7^

Furthermore, *EXT1* expression has been reported to be a promising indicator of breast cancer metastasis risk^8^ and shown to be associated with a poor prognosis in multiple myeloma.^9^

Mutations in the *EXT2* gene, located on chromosome 11, cause the type II form of multiple exostoses. In addition, different isoforms encoded by alternatively spliced transcript variants are also currently known. *EXT2* has been reported to be associated with type 2 diabetes mellitus (T2DM) in some populations^10^ as well as multiple osteochondromas,^11^ not only in humans but also in zebrafish.^12^

According to the global cancer statistics in 2018, lung cancer has the highest incidence and mortality among all tumors.^13^ Non‐small cell lung cancer (NSCLC) is the most common pathological type accounting for approximately 85% of all lung cancers.^14^ Among NSCLCs, lung squamous cell carcinoma (LUSC) is the second most common type of NSCLCs, with more than 400,000 new cases per year, and accounts for 20%–30% of NSCLCs.^15^, ^16^ Despite advances in treatment methods for LUSC, the 5‐year overall survival (OS) rate of LUSC patients in clinical stages I and II is about 40%, and that of LUSC patients in clinical stages III and IV is less than 5%.^17^ Therefore, the identification of new prognostic markers and therapeutic targets is important for the clinical treatment of LUSC.

In this study, we performed a series of bioinformatics analyses on *EXT1* and *EXT2* in LUSC, including transcriptional analysis, co‐expression analysis, functional annotation enrichment analysis, protein‐protein interaction (PPI) analysis, survival analysis, and constructed a miRNA‐EXT regulation network. We observed increased levels of *EXT1* and *EXT2* expression in LUSC, whereas only *EXT1* was associated with poor OS prognosis in LUSC. Furthermore, we identified three regulatory miRNAs of *EXT1*, hsa‐mir‐190a‐5p, hsa‐mir‐195‐5p, and hsa‐mir‐490‐3p, which could potentially be involved in molecular mechanisms underlying of the disease. Our results thus provide novel insights to improve the prognosis of LUSC patients.

## 2 MATERIALS AND METHODS

### 2.1 Bibliometric analysis

VOS viewer is primarily intended to be used for analyzing bibliometric networks.^18^ In the view, the larger the number of items in the neighborhood of a point and the higher the weights of the items, the closer the color of the point is to red.

### 2.2 Oncomine analysis

Oncomine (www.oncomine.org), a cancer microarray database and web‐based data‐mining platform, was used to analyze the transcription levels of *EXT1* and *EXT2* in different cancers. The mRNA expression of *EXT1* and *EXT2* in clinical cancer specimens were compared with that in normal controls, using the Student's *t*‐test. Fold change>1.5 with *p*‐value <0.01 was considered statistically significant.

### 2.3 UALCAN analysis

To increase the credibility of the data, we further analyzed the transcriptional and clinical data for *EXT1* and *EXT2* from TCGA. The UALCAN platform (http://ualcan.path.uab.edu) allows users to examine relative expression levels of a query gene or gene set among specified tumor sub‐groups. These pre‐defined tumor sub‐groups include cancer stage, tumor grade, race, or other clinicopathologic features.^19^

### 2.4 CCLE analysis

The Cancer Cell Line Encyclopedia (CCLE)^20^ (www.broadinstitute.org/ccle) project is a collaboration between the Broad Institute, the Novartis Institutes for Biomedical Research and the Genomics Institute of the Novartis Research Foundation to conduct a detailed genetic and pharmacologic characterization of a large panel of human cancer models, to develop integrated computational analyses that link distinct pharmacologic vulnerabilities to genomic patterns and to translate cell line integrative genomics into cancer patient stratification.^21^ CCLE is a public database that supports genomic data analysis and visualization of about 1000 cell lines. *EXT1* and *EXT2* expression in cancer cell lines was verified using the CCLE datasets.

### 2.5 Cell culture

Human NSCLC cells (A549, PC9, NCI‐H1299, NCI‐H460, NCI‐H23) and human bronchial epithelioid cells (HBE) were cultured in Dulbecco's Modified Eagle Medium with 4.5 g/L glucose (DMEM, Gibco BRL) containing 10% fetal bovine serum (FBS, Gibco BRL) and 1% antibiotic/antimycotic solution. Cells were maintained at 37°C in an atmosphere of 5% CO_2_.

### 2.6 RNA extraction and quantitative real‐time PCR

Total RNA was extracted from cells using Trizol reagent (Sangon Biotech) according to the manufacturer's instructions. For mRNAs quantification, RNA was reverse transcribed to cDNA using the PrimeScript™ RT reagent Kit with gDNA Eraser (Takara). Quantitative real‐time PCR was performed using cDNA primers specific for mRNA. All the real‐time PCR reactions were performed using Takara Bio's SYBR Premix Ex Taq™ II in the BIO‐RAD CFX96 Real‐Time PCR System. The 2^−ΔΔCt^ method was used for quantification and fold change for target genes was normalized by internal control. The PCR reaction conditions were as follows: 95°C for 10 min followed by 40 cycles of 95°C for 5 sec, 60°C for 30 sec and 72°C for 30 sec. The expression levels were normalized against those of the internal reference gene β‐actin.

The following primers were used: β‐actin forward 5′‐CCCAGCACAATGAAGATCAA‐3′ and reverse 5′‐ACATCGCTGGAAGGTGGAC‐3′; *EXT1* forward 5′‐TGCCTGTCGTCGTCATTGAA‐3′ and reverse 5′‐ACGGCGTCTGTGATGATGTT‐3′; *EXT2* forward: 5′‐TTATGTGTGCGTCGGTCAAGT‐3′ and reverse 5′‐AGGACAATGGAGAAGAGGGTG‐3′.

### 2.7 Western blot

Western blot was carried out according to previous publications.^22^ The anti‐*EXT1* (A‐7) (Santa Cruz, 1:2000), anti‐*EXT2* (A‐2) (Santa Cruz, 1:2000), anti‐Actin (Santa Cruz, 1:4000) were used as the primary antibodies. A 1:3000–5000 dilution of the HRP‐linked anti‐IgG (Santa Cruz) was used as the secondary antibody.

### 2.8 Co‐expressed genes

The top 100 genes co‐expressed genes with *EXT1* were selected from the co‐expressed genes datasets in the Oncomine database, based on a cut‐off of *p*‐value ≤0.01 and fold change ≥1.5.

### 2.9 PPI networks

The STRING (Search Tool for the Retrieval of Interacting Genes) database (https://string‐db.org, version 11.0) is a biological database designed for the construction of PPI network of genes, based on known and predicted PPIs, and analysis of the functional interactions between proteins.^23^ Analysis of the functional interactions between proteins may provide insights into the mechanisms underlying the development of diseases. In this study, a PPI network of co‐expressed genes was constructed using the STRING database and an interaction with a combined score >0.4 was considered statistically significant. Cytoscape (version 3.7.2),^24^ an open source bioinformatics software platform, was used for visualizing the molecular interaction networks.

### 2.10 GO annotation enrichment and KEGG pathway enrichment analysis

The gene ontology (GO) resource provides a platform for functional annotation and enrichment analysis of genes.^25^ KEGG (Kyoto Encyclopedia of Genes and Genomes) is a comprehensive database of biological information designed to assist in the interpretation of large‐scale molecular data sets.^26^ *p* < 0.05 was considered statistically significant for GO annotation enrichment analysis and KEGG pathway enrichment analysis.

### 2.11 ENCORI database

ENCORI (Encyclopedia of RNA Interactomes; http://starbase.sysu.edu.cn/) is an open‐source platform for studying the miRNA‐ncRNA, miRNA‐mRNA, ncRNA‐RNA, RNA‐RNA, RBP‐ncRNA, and RBP‐mRNA interactions from CLIP‐seq, degradome‐seq, and RNA‐RNA interactome data.^27^ In our study, ENCORI was used to predict miRNAs regulating *EXT1* and verify the correlation with RNA expression. The options used in the analysis were as follows: CLIP Data: high stringency (≥3), Degradome Data: with or without data Pan‐ Cancer: 1 Cancer type.

### 2.12 The Kaplan–Meier plotter

The prognostic significance of expression of identified miRNAs in LUSC was evaluated using the Kaplan–Meier plotter (www.kmplot.com), an online tool for meta‐analysis based discovery and validation of survival biomarkers with data based on gene expression and clinical data from multiple sources. To assess the prognostic value of a specific miRNA, patient samples are divided into two cohorts according to the median expression of the gene (high vs. low). We obtained the Kaplan‐Meier survival plots for the shortlisted miRNAs and assessed the association with OS in LUSC patients based on the number‐at‐risk values, log rank *p*‐value and hazard ratio (HR) with 95% confidence intervals available for each plot.

### 2.13 Identification of candidate miRNAs and miRNA‐mRNA regulation network

Although considerable progress has been made, identification of differentially expressed miRNAs involved in the regulation of mRNA is still critical for a complete understanding of miRNA‐mRNA regulation network in LUSC. We compared the transcriptional levels of miRNAs in LUSC with those in normal samples by using ENCORI database. Further, as *EXT1* overexpression was associated with unfavorable prognosis in LUSC, we hypothesize that the miRNAs regulating *EXT1* should ideally predict favorable prognosis. Predicted miRNAs‐mRNA regulation networks were visualized in Cytoscape.

## 3 RESULTS

### 3.1 *EXT1* and *EXT2* expression in LUSC patients

**FIGURE 1:**
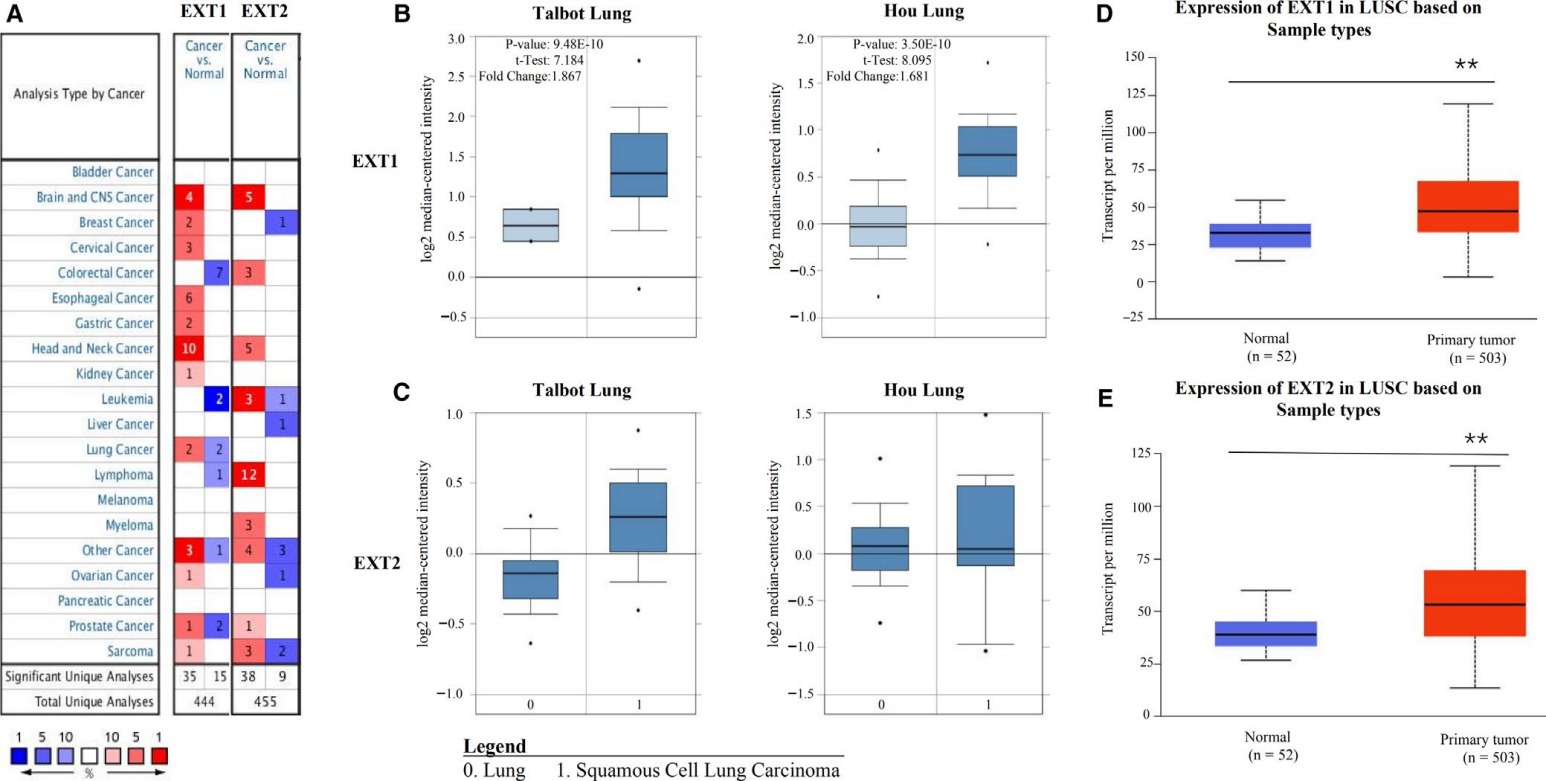
*EXT1* and *EXT2* expression in different types of cancers and LUSC (Oncomine and UALCAN). (A) Red indicates up‐regulated expression and blue indicates down‐regulated expression in the tumor tissues. Higher significance is indicated by a darker shade. The number within cells represents the number of datasets. (B and C) The expression of *EXT1* and *EXT2* in the LUSC samples in two datasets (Talbot Lung and Hou Lung). (D and E) The expression levels of *EXT1* and *EXT2* were up‐regulated in LUSC tissues. (EXT: Exostosin, LUSC: lung squamous cell carcinoma)

**FIGURE 2:**
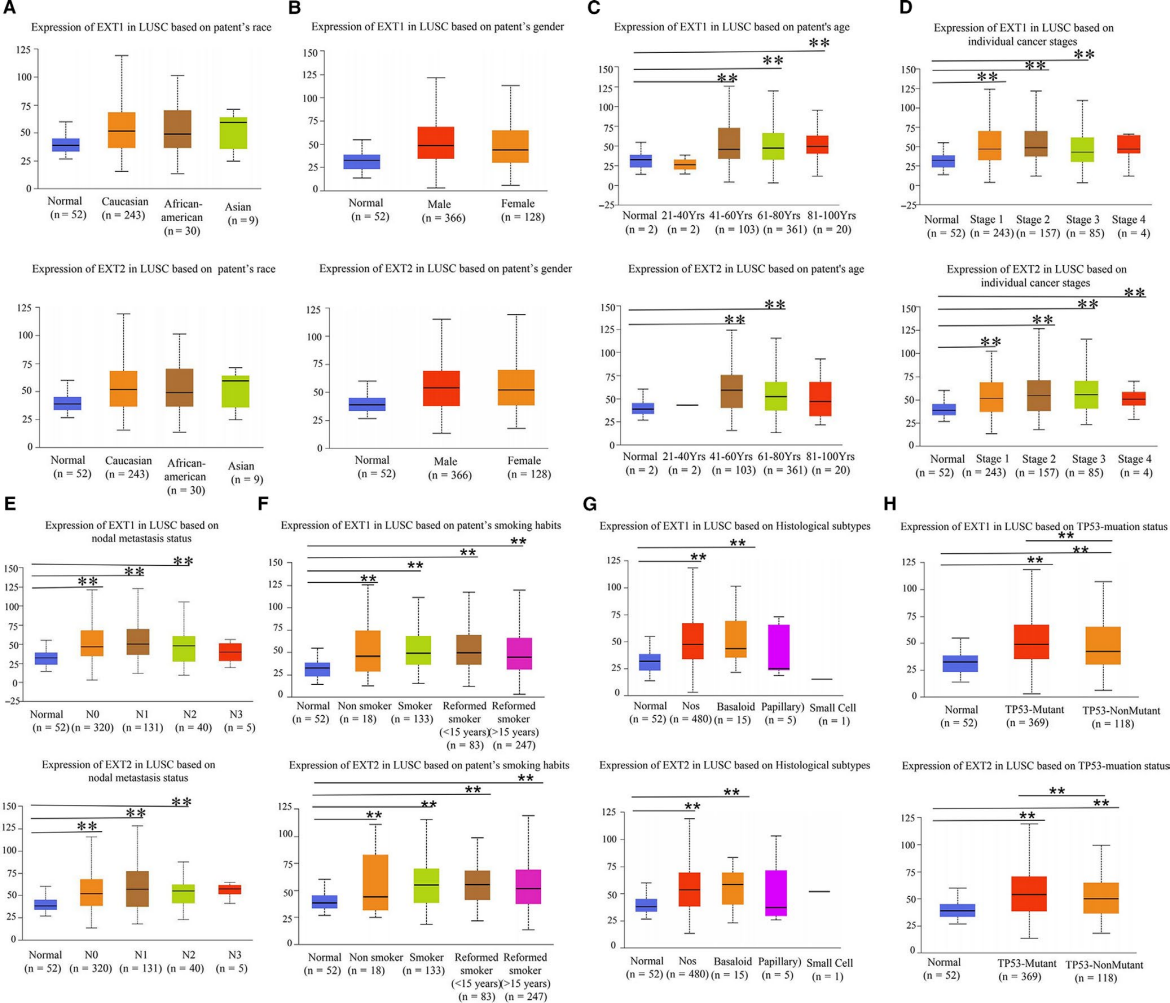
*EXT1* and *EXT2* expression in LUSC based on clinical data from the UALCAN. (A–H) *EXT1* and *EXT2* were significantly correlated with age of onset, pathological stage, lymphatic metastasis, moking habits, histological subtypes, and TP53‐muation status. (EXT, Exostosin; LUSC, lung squamous cell carcinoma)

There were 239 relevant literatures with EXT as the keyword in PubMed from 2010 to 2020. As shown in Figure S1, EXT is worth noting that tumor biomarkers are also a prominent focus of research. Nevertheless, the expression and prognostic value of EXT genes in human LUSC are not well understood. We compared the mRNA expression of *EXT1* and *EXT2* in LUSC samples with those in normal samples in the Oncomine database (Figure 1A). The expression levels of *EXT1* were significantly higher (*p* < 0.001) in two datasets (the Talbot Lung and Hou Lung) as compared with normal samples (Figure 1B). However, *EXT2* expression levels were not significantly different between tumor and normal tissues (Figure 1C). Notably, the expression of both *EXT1* and *EXT2* in LUSC tissues was significantly higher than those in normal tissues in the UALCAN analysis of samples from TCGA database (Figure 1D and E). Statistically significant differences were observed between tumor and normal samples grouped based on clinical data such as age, tumor stage, lymph node metastasis, smoking habits, histological subtypes, and TP53‐muation status (Figure 2C–H), but there were no differences in race or gender (Figure 2A and B).

### 3.2 *EXT1* and *EXT2* expression in NSCLC cell lines

**FIGURE 3:**
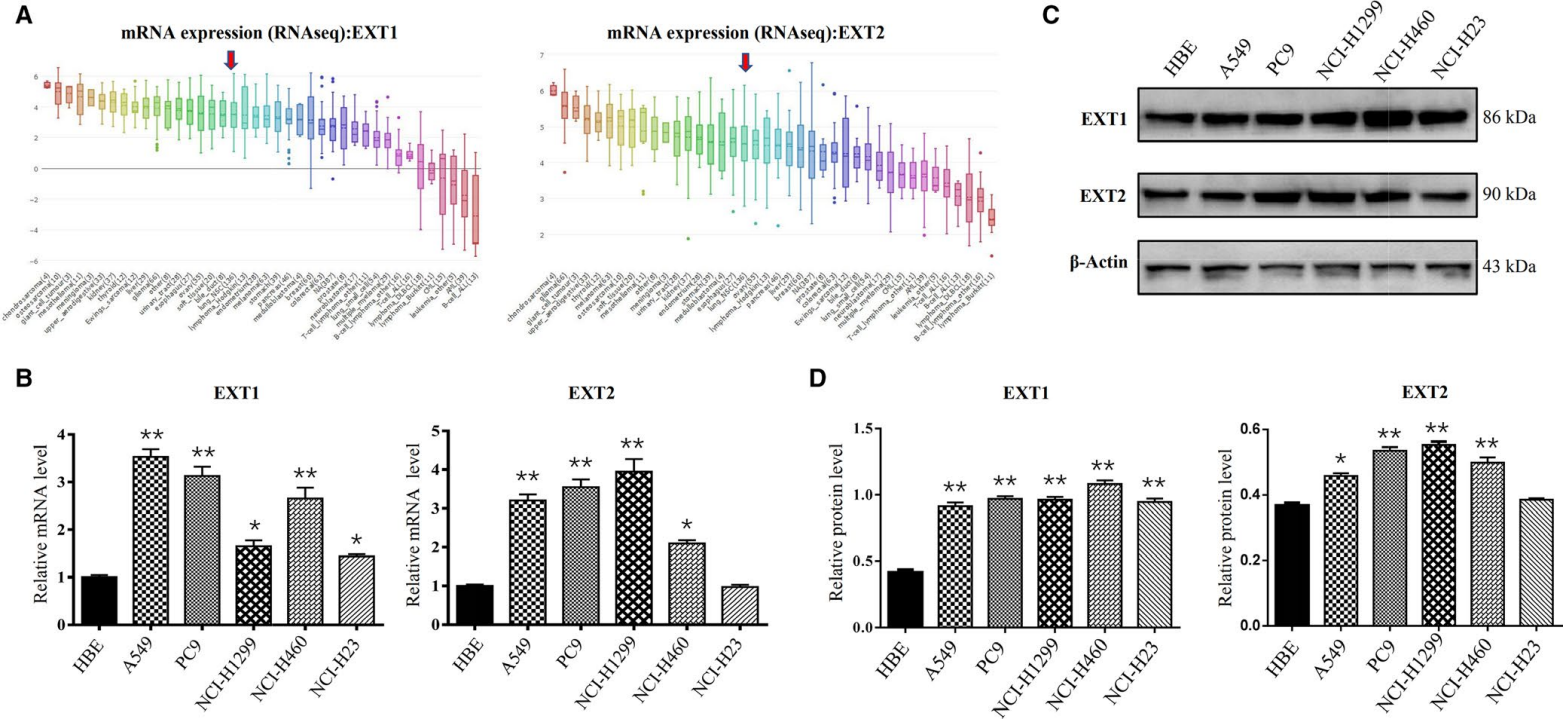
*EXT1* and *EXT2* expression in NSCLC cell lines. (A) The cell line indicated by the red arrow is Lung‐NSC. *EXT1* and *EXT2* were overexpressed in Lung‐NSC cell lines (CCLE). (B) *EXT1* and *EXT2* expression in human NSCLC cell lines. C, D: *EXT1* and *EXT2* protein expression in human NSCLC cell lines. (**p < 0.01, *p < 0.05 as compared with the HBE cell line). (EXT, Exostosin; LUSC, lung squamous cell carcinoma, NSCLC, Non‐small cell lung cancer; CCLE, Cancer Cell Line Encyclopedia)

We included data from the Cancer Cell Line Encyclopedia (CCLE) (www.broadinstitute.org/ccle) database to extend our analysis to preclinical human cancer models. We observed high expression of *EXT1* and *EXT2* in NSCLC cell lines (Figure 3A). To validate the findings from the analysis of microarray‐based datasets, we measured the expression of EXT mRNA and protein in five NSCLC cell lines (A549, PC9, NCI‐H1299, NCI‐H460, and NCI‐H23) and human bronchial epithelioid (HBE) cells by qRT‐PCR and western blot, respectively. Those results confirmed that not only *EXT1*, but also *EXT1* expression levels were significantly higher in NSCLC cell lines than those in the control HBE cells (*p* < 0.01), consistent with the results of our analysis (Figure 3B–D). Similarly, *EXT2* was also significantly overexpressed in all NSCLC cell lines (*p* < 0.01), except NCI‐H23 (Figure 3B–D). These results suggest that upregulation of *EXT1* and *EXT2* may be closely associated with the biological characteristics of malignant LUSC.

### 3.3 Association of *EXT1* and *EXT2* expression with prognosis in LUSC patients

**FIGURE 4:**
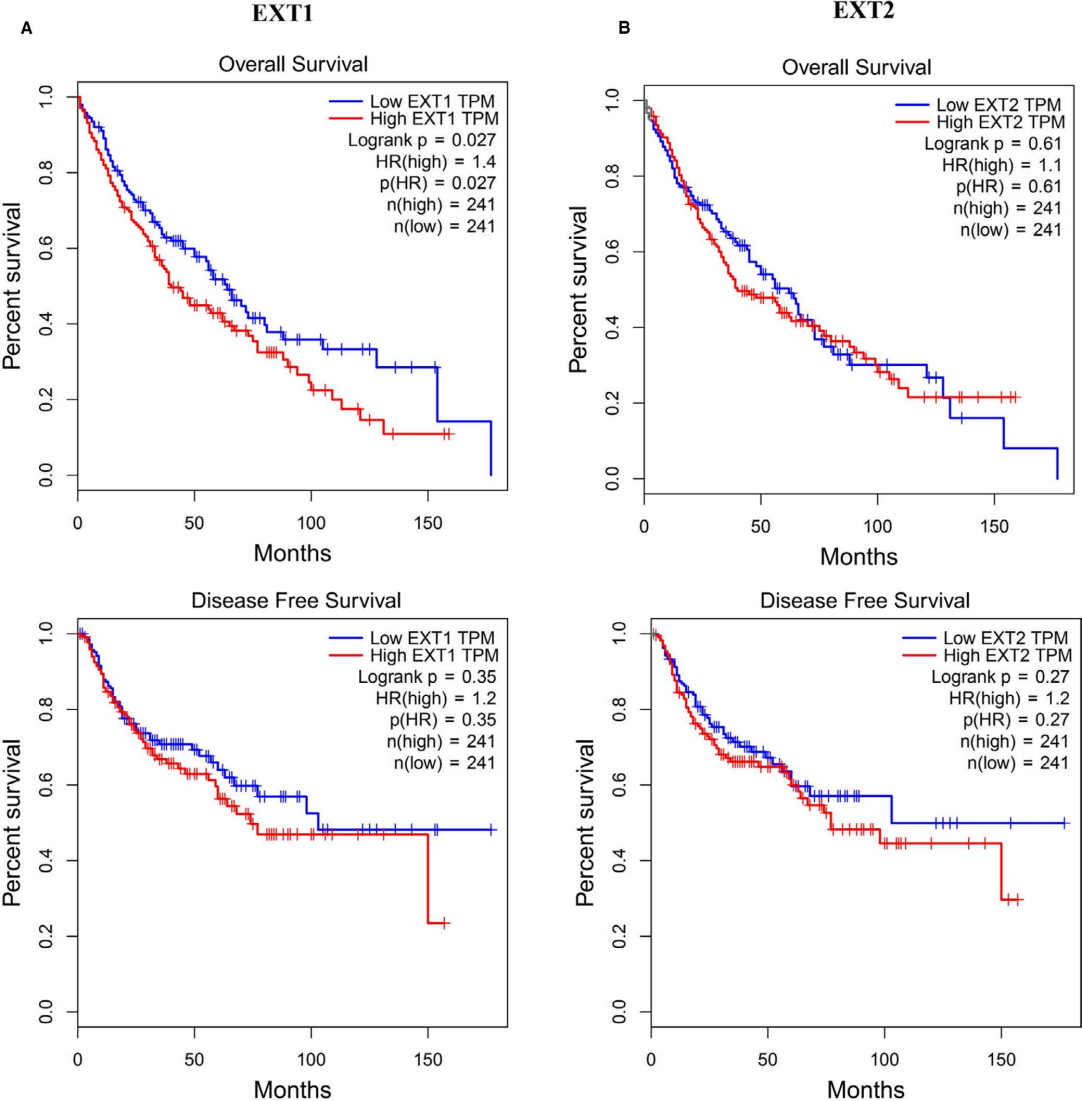
Kaplan–Meier plots for mRNA expression of *EXT1* and *EXT2* in LUSC patients (GEPIA). (A) *EXT1* overexpression was significantly associated with poor prognosis of overall survival (*p* < 0.027) but not with disease free survival. (B) *EXT2* expression was not significantly associated with prognosis. (EXT, Exostosin; LUSC, lung squamous cell carcinoma; GEPIA, Gene Expression Profiling Interactive Analysis)

The association of *EXT1* and *EXT2* expression with OS and disease‐free survival (DFS) in patients with LUSC was analyzed using the GEPIA server. As shown in Figure 4A, the OS rate of patients with high *EXT1* expression was significantly lower than that of patients with low *EXT1* expression (*p* = 0.027), but the association with DFS rate was not statistically significant (*p* = 0.35). The association of *EXT2* expression with both OS rate and DFS rate of LUSC patients was not statistically significant (Figure 4B). Thus, survival analysis revealed that increased *EXT1* mRNA levels were significantly associated with reduced OS in LUSC patients.

### 3.4 Genes co‐expressed with *EXT1* and functional enrichment analysis

**FIGURE 5:**
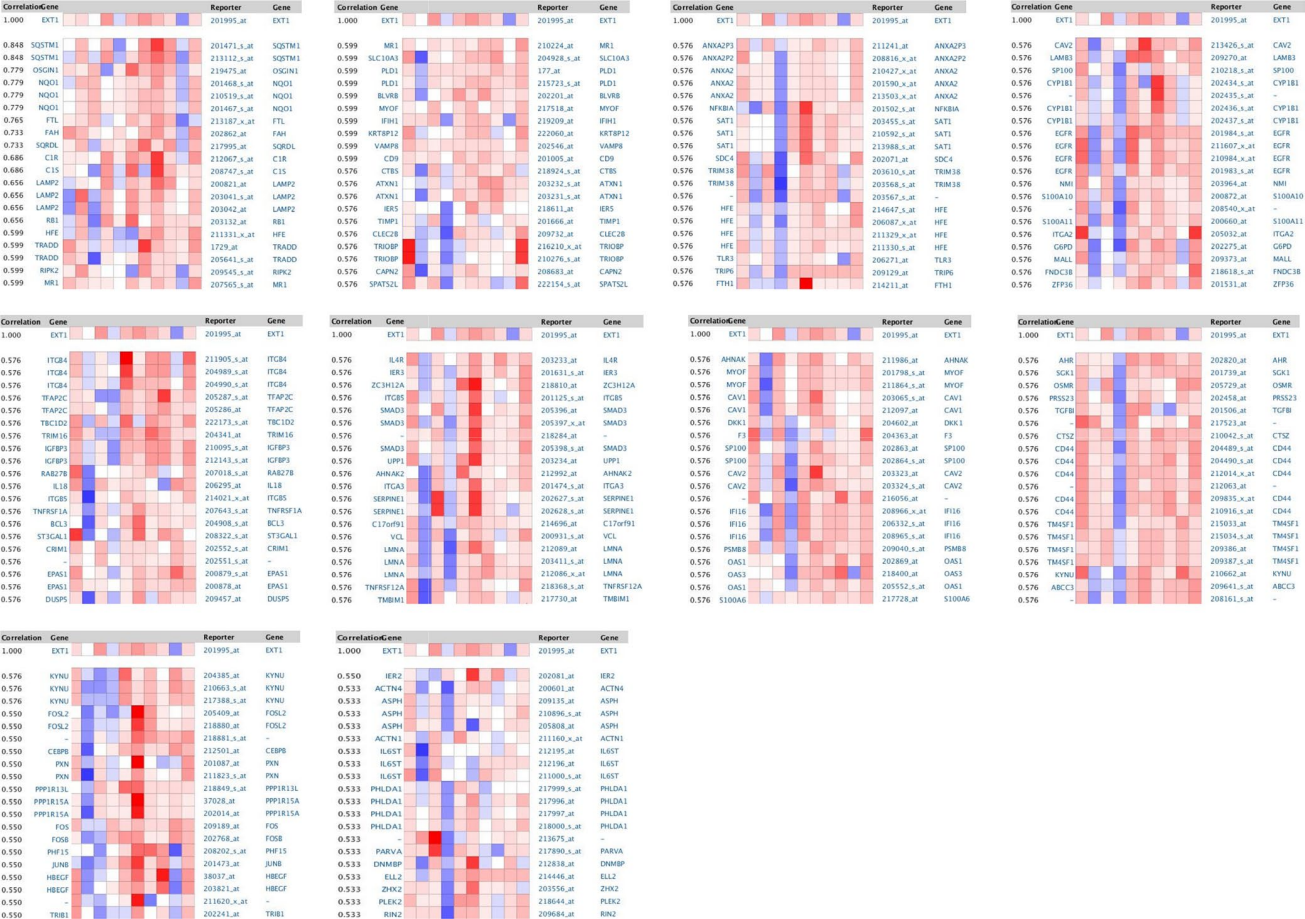
Genes co‐expressed with *EXT1*. The top 100 co‐expressed genes in LUSC were screened from the Gemma Cell Line dataset in the Oncomine database (EXT, Exostosin; LUSC, lung squamous cell carcinoma)

**FIGURE 6:**
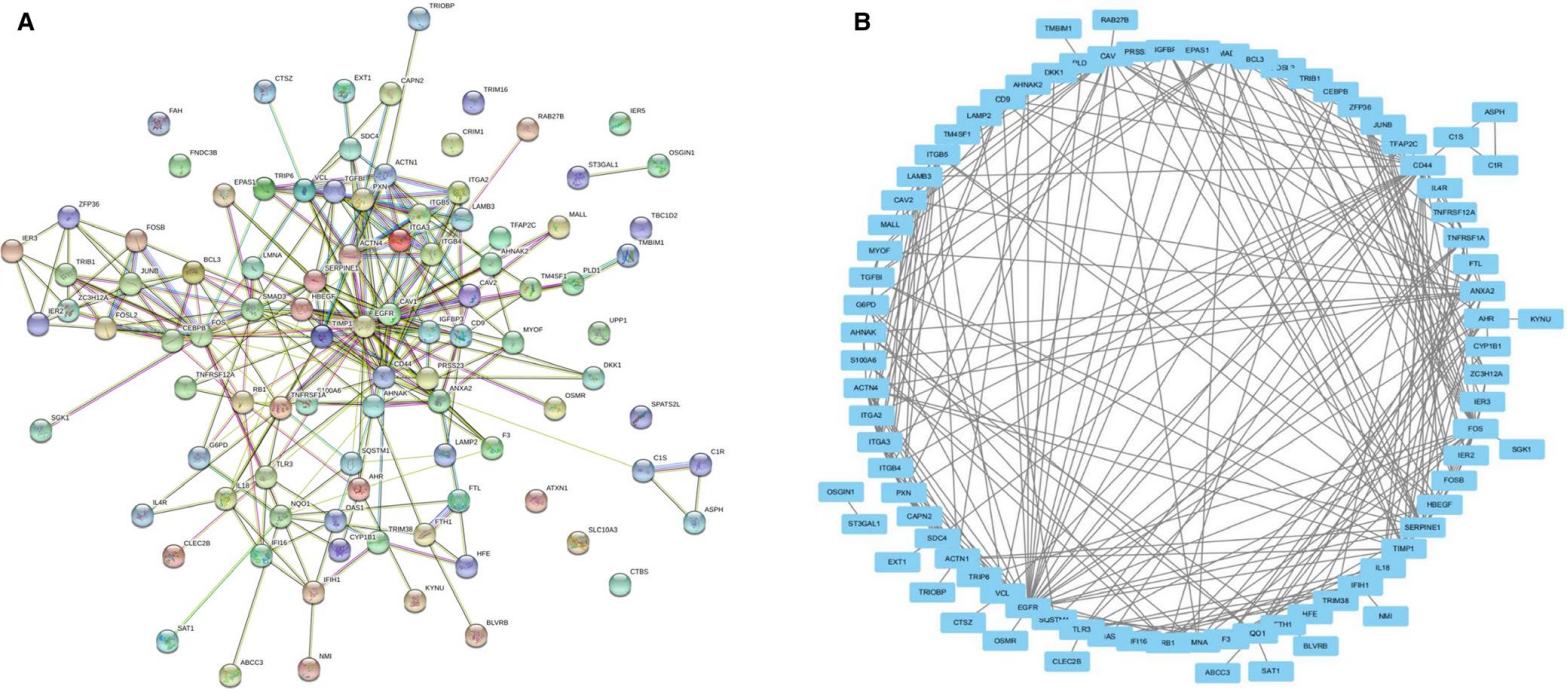
PPI network of genes co‐expressed with *EXT1* (A) PPI network of genes co‐expressed with *EXT1* constructed in the STRING database. (B) Visualization of a STRING‐derived network of molecular interactions in Cytoscape pathway visualization and analysis software (EXT1, Exostosin; PPI, protein‐protein interaction; STRING, Search Tool for the Retrieval of Interacting Genes)

**FIGURE 7:**
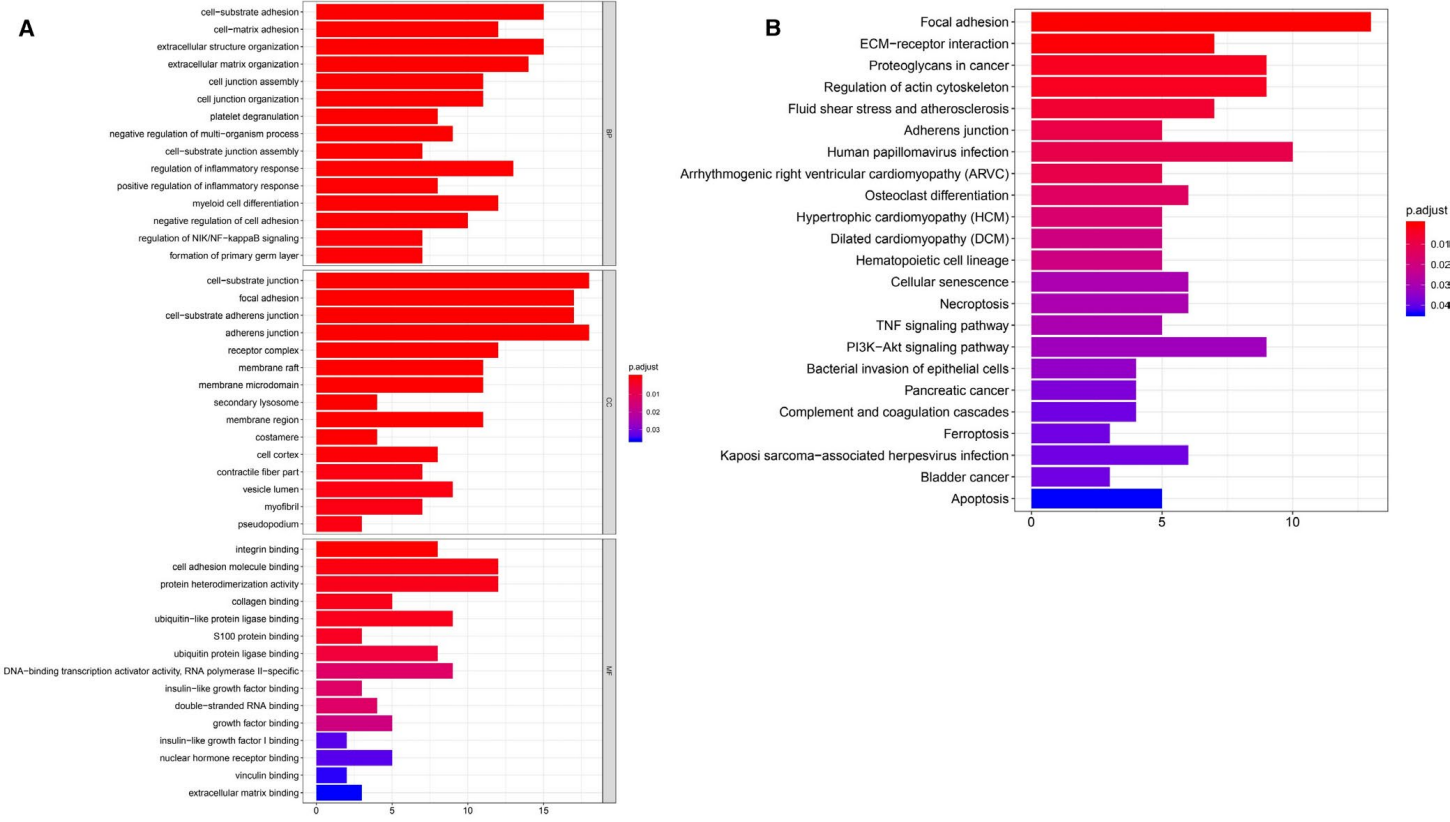
GO functional annotation enrichment and KEGG pathway enrichment analysis of genes co‐expressed with *EXT1*. (A) Co‐expressed genes were most enriched in cell adhesion and migration associated annotations (B) Co‐expressed genes were most enriched in cancer related pathways such as focal adhesion, ECM‐receptor interaction, proteoglycans in cancer, complement and coagulation cascade, tumor necrosis factor signaling pathway, cell death, etc. (EXT, Exostosin; GO, Gene Ontology; KEGG, Kyoto Encyclopedia of Genes and Genomes; LUSC, lung squamous cell carcinoma)

Based on the results of the expression and survival analysis described above, we selected *EXT1* for further bioinformatics analysis. The top 100 genes co‐expressed with the *EXT1* gene in LUSC were screened from the Gemma Cell Line dataset of Oncomine database (Figure 5). A protein‐protein interaction (PPI) network was generated in the STRING protein interaction database (Figure 6A) and imported into the bioinformatics software platform Cytoscape (Version 3.7.1) for visualization (Figure 6B) and further analysis. Functional annotation enrichment analysis using Gene Ontology (GO) (Figure 7A) and KEGG pathway enrichment analysis (Figure 7B) showed that the co‐expressed genes were significantly enriched in biological processes such as cell matrix adhesion, cell connectivity, regulation of inflammatory response, regulation of multi‐organism processes, and regulation of NIK/NF‐kappaB signaling, molecular functions such as cytokine binding, protein binding, receptor binding and matrix adhesion and cellular components such as cell matrix junction, membrane microstructural domain, receptor complex, and adhesion spot. The most enriched KEGG pathways included extracellular matrix receptor interaction, proteoglycans in cancer, complement and coagulation cascade, tumor necrosis factor signaling pathway, and cell death among others.

### 3.5 Regulatory miRNAs and survival analysis

**FIGURE 8:**
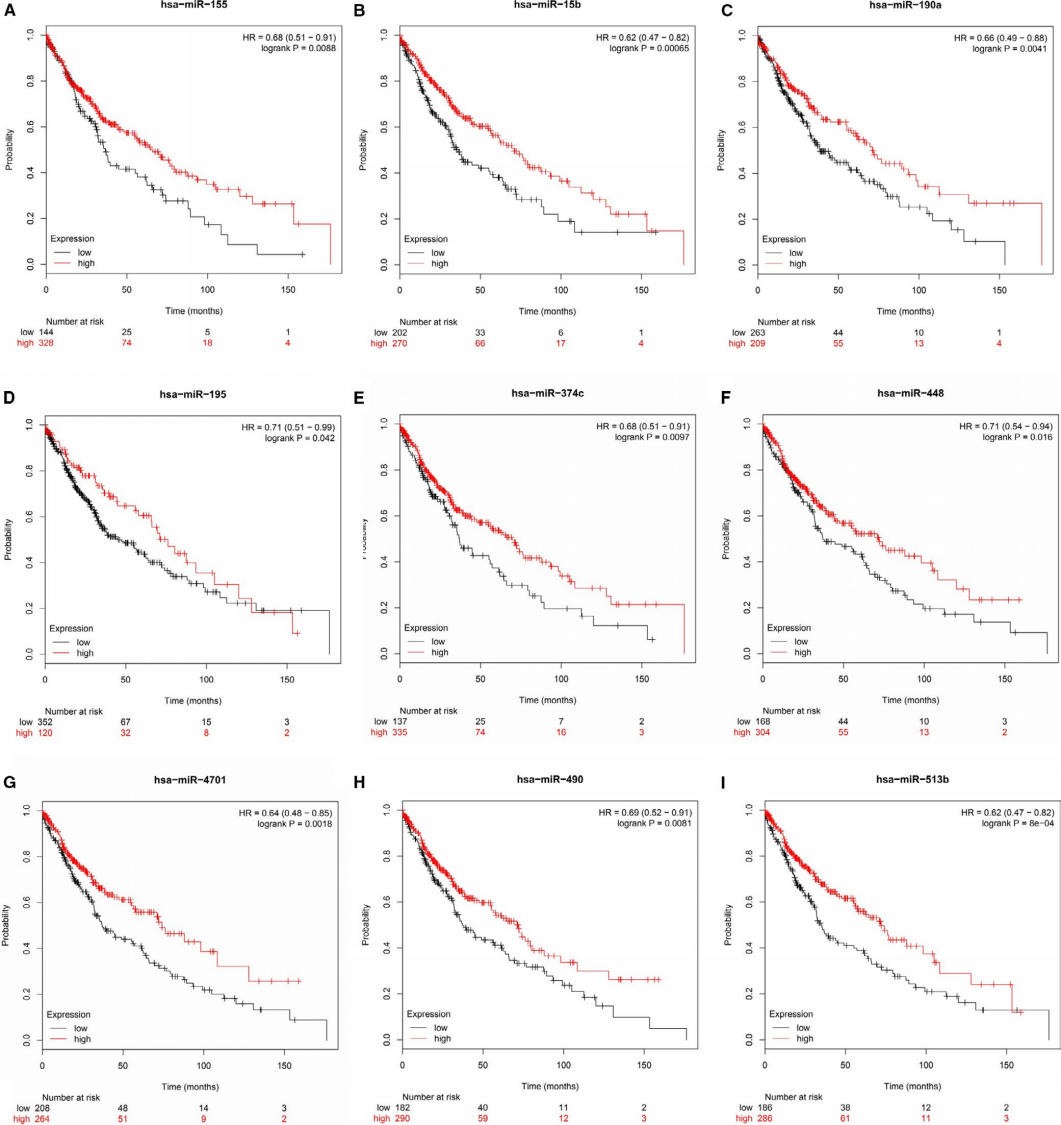
Kaplan–Meier plots for miRNAs negatively correlated with *EXT1* expression in LUSC patients (Kaplan–Meier plotter). LUSC patients with low expression of miRNAs had a poor prognosis. (EXT, Exostosin; LUSC, lung squamous cell carcinoma; miRNA, microRNA)

**FIGURE 9:**
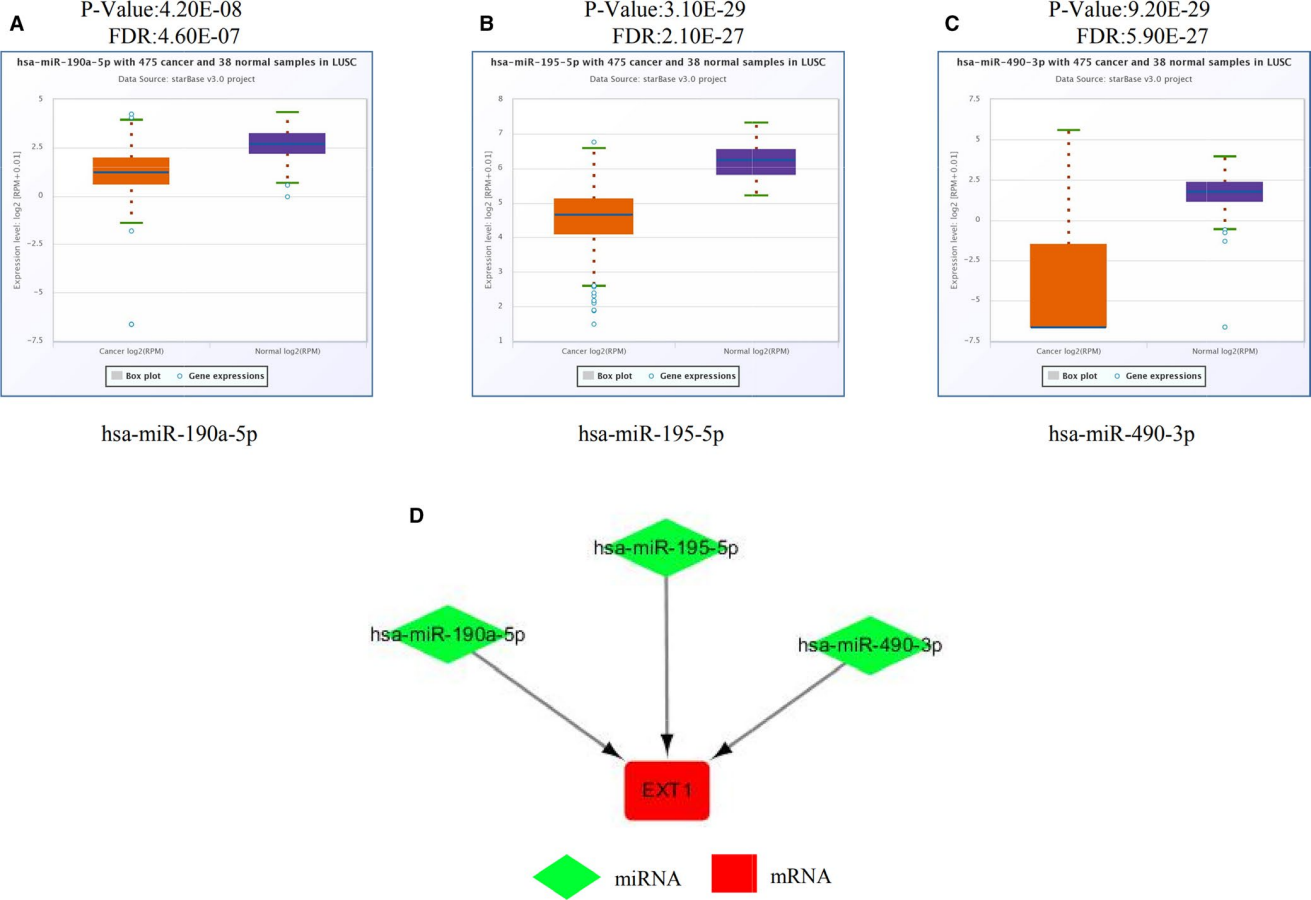
Expression of miRNAs predicted to regulate *EXT1* and miRNA‐*EXT1* regulation network (A–C) The expression levels of candidate miRNAs was down‐regulated in tumor tissues (ENCORI). (D) miRNA‐*EXT1* regulation network (Cytoscape). The expression of miRNAs (indicated in green) is down‐regulated and the expression of *EXT1* mRNA (indicated in red) is up‐regulated in LUSC. (ENCORI, Encyclopedia of RNA Interactomes; EXT, Exostosin; LUSC, lung squamous cell carcinoma; miRNA, microRNA)

MiRNAs are short non‐coding RNAs that induce mRNA silencing and destabilization by binding to specific target sites.^28^ MiRNAs inversely regulate their target mRNAs resulting in a negative correlation between miRNA and mRNA expression.^29^ Therefore, potential regulatory miRNAs should meet the following two criteria, decreased expression in LUSC samples and association of decreased expression with poor prognosis in LUSC patients. The ENCORI platform predicted a total of 42 miRNAs regulating *EXT1* (Table 1). Among them, 22 miRNA‐*EXT1* pairs were negatively correlated. The Kaplan–Meier plotter was used to evaluate the prognostic value of the 22 miRNAs in LUSC. Of these, the prediction of poor prognosis for low expression in LUSC patients was significant for nine miRNAs (Figure 8). The ENCORI pan‐cancer analysis platform was used to compare the expression of these nine miRNAs in LUSC and normal samples. Three miRNAs (hsa‐miR‐190a‐5p, hsa‐miR‐195‐5p, and hsa‐miR‐490‐3p) were found to be significantly downregulated in LUSC samples (Figure 9A–C).

**TABLE 1:** Correlation between miRNA‐EXT1 pairs identified by ENCORI database

No.	miRNA	Coefficient‐R	*p*‐Value
1	hsa‐miR‐126‐5p	−0.153	8.47E‐04
2	hsa‐miR‐153‐3p	−0.013	7.82E‐01
3	hsa‐miR‐155‐5p	−0.111	1.53E‐02
4	hsa‐miR‐15b‐5p	−0.025	5.83E‐01
5	hsa‐miR‐16‐5p	−0.171	1.75E‐04
6	hsa‐miR‐190a‐5p	−0.017	7.18E‐01
7	hsa‐miR‐190b	−0.173	1.55E‐04
8	hsa‐miR‐195‐5p	−0.025	5.94E‐01
9	hsa‐miR‐200c‐3p	−0.024	6.01E‐01
10	hsa‐miR‐3064‐5p	−0.002	9.71E‐01
11	hsa‐miR‐374c‐5p	−0.085	6.44E‐02
12	hsa‐miR‐375	−0.348	5.50E‐15
13	hsa‐miR‐448	−0.032	4.91E‐01
14	hsa‐miR‐4701‐5p	−0.027	5.63E‐01
15	hsa‐miR‐488‐3p	−0.061	1.85E‐01
16	hsa‐miR‐490‐3p	−0.13	4.64E‐03
17	hsa‐miR‐513b‐5p	−0.018	7.00E‐01
18	hsa‐miR‐514a‐5p	−0.011	8.12E‐01
19	hsa‐miR‐579‐3p	−0.005	9.10E‐01
20	hsa‐miR‐580‐3p	−0.083	7.02E‐02
21	hsa‐miR‐616‐3p	−0.092	4.41E‐02
22	hsa‐miR‐664b‐3p	−0.015	7.52E‐01
23	hsa‐miR‐129‐5p	0.071	1.20E‐01
24	hsa‐miR‐149‐5p	0.312	3.32E‐12
25	hsa‐miR‐15a‐5p	0.003	9.56E‐01
26	hsa‐miR‐199a‐5p	0.169	2.10E‐04
27	hsa‐miR‐199b‐5p	0.213	2.78E‐06
28	hsa‐miR‐200b‐3p	0.016	7.30E‐01
29	hsa‐miR‐28‐5p	0.028	5.49E‐01
30	hsa‐miR‐3140‐3p	0.067	1.42E‐01
31	hsa‐miR‐339‐5p	0.004	9.24E‐01
32	hsa‐miR‐382‐3p	0.228	4.98E‐07
33	hsa‐miR‐429	0.005	9.07E‐01
34	hsa‐miR‐4524a‐5p	0.084	6.70E‐02
35	hsa‐miR‐455‐3p	0.199	1.25E‐05
36	hsa‐miR‐4766‐3p	0.036	4.29E‐01
37	hsa‐miR‐503‐5p	0.003	9.47E‐01
38	hsa‐miR‐588	0.026	5.72E‐01
39	hsa‐miR‐655‐3p	0.132	3.92E‐3
40	hsa‐miR‐665	0.188	3.90E‐05
41	hsa‐miR‐708‐5p	0.157	5.82E‐04
42	hsa‐miR‐944	0.369	9.47E‐17

### 3.6 MiRNA‐ *EXT1* regulation network

We established a potential miRNA‐*EXT1* regulation network based on the regulatory miRNAs of *EXT1* identified by bioinformatics analysis using the ENCORI database and visualized it in Cytoscape (Figure 9D). Thus, the establishment of a potential regulatory network of miRNA‐ *EXT1* may be prognostics biomarkers and a therapeutic target.

## 4 DISCUSSION

Dysregulation of the *EXT1* gene has been reported in many cancers, including multiple osteochondroma (MO),^30^ breast cancer,^7^ ALL^31^ and HCC.^6^ To the best of our knowledge, the association of *EXT1* expression with LUSC has not been reported. This is the first study to explore the prognostic value of *EXT1* mRNA expression in LUSC. Our findings add to the current knowledge and may contribute towards improving treatment options and increase the accuracy of prognosis for patients with LUSC. It is reported that 70% to 90% MO cases are caused by pathogenic mutations in the *EXT1* or *EXT2* genes, and *EXT1* is more frequently mutated than the *EXT2* gene.^32^ Furthermore, EXT1 regulates the NOTCH pathway in an FBXW7‐dependent manner in ALL.^5^ Moreover *EXT1*‐dependent HS structure is involved in modifying tumor‐stroma interactions through altering stromal TGF‐ß1 expression in human A549 carcinoma cells.^33^

Our study of transcriptional data from Oncomine, UALCAN, TCGA and CCLE revealed increased levels of *EXT1* and *EXT2* expression in LUSC samples and cell lines. There were significant differences between tumor and normal samples grouped in age, tumor stage, lymph node metastasis, smoking habits, histological subtypes, and TP53‐muation status. Notably, the difference in expression levels between cancer and adjacent normal tissues was statistically significant only for of *EXT1* in the Talbot Lung and Hou Lung datasets. Furthermore, *EXT1* mRNA and protein expression was significantly overexpressed in the five NSCLC cell lines studied (A549, PC9, NCI‐H1299, NCI‐H460, NCI‐H23), as compared with HBE cells, whereas *EXT2* mRNA and protein expression was significantly overexpressed in all except the NCI‐H23 cell line. Survival analysis showed that patients with high *EXT1* expression had unfavorable OS prognosis. These results suggest that the overexpression of *EXT1* could be a novel potential prognostic marker in LUSC.

We mapped the top 100 genes co‐expressed with *EXT1* into the STRING database and obtained the PPI network to identify the interactions between these genes. A functional enrichment and analyze was carried out to further understand the role of genes co‐expressed with *EXT1* in LUSC. The GO enrichment analysis results indicated that these genes are primarily involved in biological processes such as cell adhesion and migration. Furthermore, KEGG pathway enrichment analysis revealed that the co‐expressed genes were enriched in multiple pathways including, extracellular matrix‐receptor interaction, proteoglycans in cancer, the complement and coagulation cascade, tumor necrosis factor signaling pathway, and cell death, among others. In particular, GO and pathway enrichment analysis indicated that the co‐expressed genes were significantly enriched in focal adhesion. It is well documented that focal adhesion and cell adhesion play a key role in cancer invasion and metastasis.^34^, ^35^ Thus, our findings show that *EXT1* may be involved in the invasion and metastasis of LUSC.

MicroRNAs (miRNAs) are short non‐coding RNAs with regulatory functions in various biological processes including cell differentiation, development and oncogenic transformation.^36^ Numerous studies have shown that miRNAs bind to the mRNA transcripts of protein‐coding genes, inhibiting their translation or leading to mRNA degradation. We used the ENCORI platform to predict the miRNAs regulating *EXT1* and found 42 miRNAs, listed in Table 1, of which 22 were down‐regulated in LUSC. Furthermore, we analyzed OS and DFS associated with the expression of these 22 miRNAs. Negatively regulated miRNA‐mRNA pairs have been reported to significantly contribute to the initialization and development of different types of cancers.^37^, ^38^, ^39^ We identified three significantly down‐regulated miRNAs, hsa‐miR‐190a‐5p, hsa‐miR‐195‐5p, and hsa‐miR‐490‐3p, with good prognostic value.

Functionally, hsa‐miR‐190a‐5p has been reported to act as a tumor suppressor in multiple malignancies. miR‐190a‐5p expression levels are significantly decreased in the cancer group compared with the normal group, and overexpression of miR‐190a‐5p inhibits cell proliferation and invasion and promotes apoptosis in cancers such as cervical cancer, neuroblastoma, and breast tumors.^40^, ^41^, ^42^ A recent study showed that smoking‐induced dysregulation of hsa‐miR‐190a‐5p was significantly associated with epithelial‐mesenchymal transition (EMT) and carcinogenesis.^43^

Furthermore, hsa‐miR‐195‐5p has also been demonstrated as a tumor suppressor in many human cancers, including renal cell carcinoma, gastric cancer, ovarian cancer, pancreatic cancer, melanoma, HCC, and colorectal cancer.^44^, ^45^, ^46^, ^47^, ^48^, ^49^, ^50^ The expression of miR‐195‐5p is decreased in NSCLC tissues and cell lines and significantly associated with the TNM stage, tumor size and lymph node metastasis, while being correlated with poor prognosis in NSCLC patients. Functional analysis has revealed that overexpression of miR‐195‐5p suppressed cell proliferation, promoted cell cycle arrest and apoptosis in NSCLC significantly.^51^

Several studies have also demonstrated similar behavior for hsa‐miR‐490‐3p, wherein decreased expression of the miRNA was significantly associated with tumorigenesis of human cancers, such as ovarian carcinoma,^52^ colorectal cancer,^53^, ^54^ glioma,^55^ prostate cancer,^56^ esophageal squamous cell carcinoma,^57^ HCC ^58^ and increased expression of the miRNA inhibited cellular growth, suppressed cellular migration and invasion.

Overall, our findings are consistent with previous studies and indicate that the three miRNAs identified in this study, hsa‐miR‐190a‐5p, hsa‐miR‐195‐5p and hsa‐miR‐490‐3p, play an important role in the inhibition of malignant tumors. Thus, we have established a potential miRNAs‐*EXT1* regulation network that may be associated with prognosis in LUSC.

In summary, based on the bioinformatics analyses presented in this study, we suggest *EXT1* as a novel potential prognostic marker for LUSC and present the miRNAs regulating *EXT1* which could be involved in carcinogenesis. We hope that our findings will benefit future studies and improve the prognosis of LUSC patients.

## CONFLICT OF INTEREST

The authors declare that the research was conducted in the absence of any commercial or financial relationships that could be construed as a potential conflict of interest.

## AUTHORS’ CONTRIBUTIONS

Disheng WU, Bilian XU, and Yi LIU contributed to study design/planning. Disheng WU, Chao HUO, Siyu JIANG, Yanxia HUANG, Xuehong FANG, Jun LIU, and Min YANG contributed to data collection/entry. Disheng WU, Chao HUO, Yanxia HUANG, and Siyu JIANG contributed to data analysis/statistics. Bilian XU and Yi LIU contributed to data interpretation. Jianwei REN, Bilian XU, and Yi LIU contributed to preparation of manuscript. Disheng WU, Chao HUO, and Siyu JIANG contributed to literature analysis/search. The authors read and approved the final manuscript.

## FUNDING INFORMATION

National Natural Science Foundation of China, Grant/Award Number: 81373499.

## ETHICAL APPROVAL

Because animal and human experiments were not involved in this study, there was no ethical statement.

## Supporting information

Fig S1

## Data Availability

The data of *EXT1* and *EXT2* levels in tissue and cell are available from Oncomine database (https://www.oncomine.org/), UALCAN database (http://ualcan.path.uab.edu/index.html) and CCLE (www.broadinstitute.org/ccle), respectively. The correlation between *EXT* and LUSC patient's over survival (OS) and disease‐free survival (DFS) are obtained from GEPIA (http://gepia.cancer‐pku.cn/) and the clinical pathological factor data can be queried from UALCAN database. The co‐expressed genes with *EXT1* are available from the Oncomine database's Gemma cell line dataset. The miRNAs regulates *EXT1* and theirs tissue expression level are available from ENCORI (http://starbase.sysu.edu.cn/). The prognostic values of miRNAs are obtained from Kaplan‐Meier plotter (https://kmplot.com/). The qPCR and western blot data used to support the findings of this study are available within the article and from the corresponding author upon request.

## References

[1] Meneghetti MC , Hughes AJ , Rudd TR , et al. Heparan sulfate and heparin interactions with proteins. J R Soc Interface. 2015;12(110):20150589. 26289657 10.1098/rsif.2015.0589 PMC4614469

[2] Li JP , Kusche‐Gullberg M . Biosynthesis, structure, and function. Int Rev Cell Mol Biol. 2016;325:215‐273. 27241222 10.1016/bs.ircmb.2016.02.009

[3] Busse‐Wicher M , Wicher KB , Kusche‐Gullberg M . The exostosin family: proteins with many functions. Matrix Biol. 2014;35:25‐33. 24128412 10.1016/j.matbio.2013.10.001

[4] Lind T , Tufaro F , McCormick C , Lindahl U , Lidholt K . The putative tumor suppressors EXT1 and EXT2 are glycosyltransferases required for the biosynthesis of heparan sulfate. J Biol Chem. 1998;273(41):26265‐26268. 9756849 10.1074/jbc.273.41.26265

[5] Daakour S , Hajingabo LJ , Kerselidou D , et al. Systematic interactome mapping of acute lymphoblastic leukemia cancer gene products reveals EXT‐1 tumor suppressor as a Notch1 and FBWX7 common interactor. BMC Cancer. 2016;16:335. 27229929 10.1186/s12885-016-2374-2 PMC4882867

[6] Dong S , Wu Y , Yu S , Yang Y , Lu L , Fan S . Increased EXT1 gene copy number correlates with increased mRNA level predicts short disease‐free survival in hepatocellular carcinoma without vascular invasion. Medicine. 2018;97(39):e12625. 30278583 10.1097/MD.0000000000012625 PMC6181523

[7] Sembajwe LF , Katta K , Gronning M , Kusche‐Gullberg M . The exostosin family of glycosyltransferases: mRNA expression profiles and heparan sulphate structure in human breast carcinoma cell lines. Biosci Rep. 2018;38(4).BSR20180770 30054430 10.1042/BSR20180770 PMC6117623

[8] Taghavi A , Akbari ME , Hashemi‐Bahremani M , et al. Gene expression profiling of the 8q22‐24 position in human breast cancer: TSPYL5, MTDH, ATAD2 and CCNE2 genes are implicated in oncogenesis, while WISP1 and EXT1 genes may predict a risk of metastasis. Oncol Lett. 2016;12(5):3845‐3855. 27895739 10.3892/ol.2016.5218 PMC5104179

[9] Bret C , Hose D , Reme T , et al. Expression of genes encoding for proteins involved in heparan sulphate and chondroitin sulphate chain synthesis and modification in normal and malignant plasma cells. Br J Haematol. 2009;145(3):350‐368. 19298595 10.1111/j.1365-2141.2009.07633.x PMC2730414

[10] Nemr R , Al‐Busaidi AS , Sater MS , et al. Lack of replication of common EXT2 gene variants with susceptibility to type 2 diabetes in Lebanese Arabs. Diabetes Metab. 2013;39(6):532‐536. 23871501 10.1016/j.diabet.2013.05.001

[11] Chen Z , Bi Q , Kong M , Cao L , Ruan W . A novel EXT2 frameshift mutation identified in a family with multiple osteochondromas. Oncol Lett. 2018;16(4):5167‐5171. 30250583 10.3892/ol.2018.9248 PMC6144921

[12] Wiweger MI , de Andrea CE , Scheepstra KW , Zhao Z , Hogendoorn PC . Possible effects of EXT2 on mesenchymal differentiation–lessons from the zebrafish. Orphanet J Rare Dis. 2014;9:35. 24628984 10.1186/1750-1172-9-35 PMC4004154

[13] Bray F , Ferlay J , Soerjomataram I , Siegel RL , Torre LA , Jemal A . Global cancer statistics 2018: GLOBOCAN estimates of incidence and mortality worldwide for 36 cancers in 185 countries. CA Cancer J Clin. 2018;68(6):394‐424. 30207593 10.3322/caac.21492

[14] Blandin Knight S , Crosbie PA , Balata H , Chudziak J , Hussell T , Dive C . Progress and prospects of early detection in lung cancer. Open Biol. 2017;7(9):170070. 28878044 10.1098/rsob.170070 PMC5627048

[15] Ling DJ , Chen ZS , Liao QD , Feng JX , Zhang XY , Yin TY . Differential effects of MTSS1 on invasion and proliferation in subtypes of non‐small cell lung cancer cells. Exp Ther Med. 2016;12(2):1225‐1231. 27446348 10.3892/etm.2016.3382 PMC4950550

[16] Gandara DR , Hammerman PS , Sos ML , Lara PN Jr , Hirsch FR . Squamous cell lung cancer: from tumor genomics to cancer therapeutics. Clin Cancer Res. 2015;21(10):2236‐2243. 25979930 10.1158/1078-0432.CCR-14-3039 PMC4862209

[17] Tanoue LT , Detterbeck FC . New TNM classification for non‐small‐cell lung cancer. Expert Rev Anticancer Ther. 2009;9(4):413‐423. 19374596 10.1586/era.09.11

[18] van Eck NJ , Waltman L . Software survey: VOSviewer, a computer program for bibliometric mapping. Scientometrics. 2010;84(2):523‐538. 20585380 10.1007/s11192-009-0146-3 PMC2883932

[19] Chandrashekar DS , Bashel B , Balasubramanya SAH , et al. UALCAN: a portal for facilitating tumor subgroup gene expression and survival analyses. Neoplasia. 2017;19(8):649‐658. 28732212 10.1016/j.neo.2017.05.002 PMC5516091

[20] Ghandi M , Huang FW , Jane‐Valbuena J , et al. Next‐generation characterization of the Cancer Cell Line Encyclopedia. Nature. 2019;569(7757):503‐508. 31068700 10.1038/s41586-019-1186-3 PMC6697103

[21] Barretina J , Caponigro G , Stransky N , et al. The Cancer Cell Line Encyclopedia enables predictive modelling of anticancer drug sensitivity. Nature. 2012;483(7391):603‐607. 22460905 10.1038/nature11003 PMC3320027

[22] Liu Y , Yang S , Li MY , et al. Tumorigenesis of smoking carcinogen 4‐(methylnitrosamino)‐1‐(3‐pyridyl)‐1‐butanone is related to its ability to stimulate thromboxane synthase and enhance stemness of non‐small cell lung cancer stem cells. Cancer Lett. 2016;370(2):198‐206. 26518146 10.1016/j.canlet.2015.10.017

[23] Szklarczyk D , Gable AL , Lyon D , et al. STRING v11: protein‐protein association networks with increased coverage, supporting functional discovery in genome‐wide experimental datasets. Nucleic Acids Res. 2019;47(D1):D607‐D613. 30476243 10.1093/nar/gky1131 PMC6323986

[24] Doncheva NT , Morris JH , Gorodkin J , Jensen LJ . Cytoscape StringApp: network Analysis and visualization of proteomics data. J Proteome Res. 2019;18(2):623‐632. 30450911 10.1021/acs.jproteome.8b00702 PMC6800166

[25] The Gene Ontology Resource: 20 years and still GOing strong. Nucleic Acids Research. 2019;47 (D1):D330–D338. 10.1093/nar/gky1055 30395331 PMC6323945

[26] Kanehisa M , Furumichi M , Tanabe M , Sato Y , Morishima K . KEGG: new perspectives on genomes, pathways, diseases and drugs. Nucleic Acids Res. 2017;45(D1):D353‐D361. 27899662 10.1093/nar/gkw1092 PMC5210567

[27] Li JH , Liu S , Zhou H , Qu LH , Yang JH . starBase v2.0: decoding miRNA‐ceRNA, miRNA‐ncRNA and protein‐RNA interaction networks from large‐scale CLIP‐Seq data. Nucleic Acids Res. 2014;42(Database:issue):D92‐D97. 24297251 10.1093/nar/gkt1248 PMC3964941

[28] Tat TT , Maroney PA , Chamnongpol S , Coller J , Nilsen TW . Cotranslational microRNA mediated messenger RNA destabilization. eLife. 2016;5 10.7554/elife.12880 PMC4859803 27058298

[29] Diaz G , Zamboni F , Tice A , Farci P . Integrated ordination of miRNA and mRNA expression profiles. BMC Genomics. 2015;16:767. 26459852 10.1186/s12864-015-1971-9 PMC4603994

[30] Chen Z , Bi Q , Kong M , Chen Y . A Novel EXT1 mutation identified in a family with multiple osteochondromas. Genet Test Mol Biomarkers. 2019;23(4):251‐254. 29989442 10.1089/gtmb.2018.0072

[31] Liu NW , Huang X , Liu S , Lu Y . EXT1, Regulated by MiR‐665, promotes cell apoptosis via ERK1/2 signaling pathway in acute lymphoblastic leukemia. Med Sci Monit. 2019;25:6491‐6503. 31465316 10.12659/MSM.918295 PMC6733154

[32] Santos SCL , Rizzo I , Takata RI , Speck‐Martins CE , Brum JM , Sollaci C . Analysis of mutations in EXT1 and EXT2 in Brazilian patients with multiple osteochondromas. Mol Genet Genomic Med. 2018;6(3):382‐392. 29529714 10.1002/mgg3.382 PMC6014457

[33] Katta K , Sembajwe LF , Kusche‐Gullberg M . Potential role for Ext1‐dependent heparan sulfate in regulating P311 gene expression in A549 carcinoma cells. Biochim Biophys Acta Gen Subj. 2018;1862(6):1472‐1481. 29580921 10.1016/j.bbagen.2018.03.024

[34] Duperret EK , Dahal A , Ridky TW . Focal‐adhesion‐independent integrin‐alphav regulation of FAK and c‐Myc is necessary for 3D skin formation and tumor invasion. J Cell Sci. 2015;128(21):3997‐4013. 26359297 10.1242/jcs.175539 PMC4647167

[35] Eke I , Cordes N . Focal adhesion signaling and therapy resistance in cancer. Semin Cancer Biol. 2015;31:65‐75. 25117005 10.1016/j.semcancer.2014.07.009

[36] Krol J , Loedige I , Filipowicz W . The widespread regulation of microRNA biogenesis, function and decay. Nat Rev Genet. 2010;11(9):597‐610. 20661255 10.1038/nrg2843

[37] Luo D , Wilson JM , Harvel N , et al. A systematic evaluation of miRNA:mRNA interactions involved in the migration and invasion of breast cancer cells. J Transl Med. 2013;11:57. 23497265 10.1186/1479-5876-11-57 PMC3599769

[38] Zhou X , Xu X , Wang J , Lin J , Chen W . Identifying miRNA/mRNA negative regulation pairs in colorectal cancer. Sci Rep. 2015;5:12995. 26269151 10.1038/srep12995 PMC4534763

[39] Andres‐Leon E , Cases I , Alonso S , Rojas AM . Novel miRNA‐mRNA interactions conserved in essential cancer pathways. Sci Rep. 2017;7:46101. 28387377 10.1038/srep46101 PMC5384238

[40] Han MS , Lee JM , Kim SN , Kim JH , Kim HS . Human Papillomavirus 16 oncoproteins downregulate the expression of miR‐148a‐3p, miR‐190a‐5p, and miR‐199b‐5p in cervical cancer. Biomed Res Int. 2018;2018:1942867. 30627542 10.1155/2018/1942867 PMC6304571

[41] Almog N , Briggs C , Beheshti A , et al. Transcriptional changes induced by the tumor dormancy‐associated microRNA‐190. Transcription. 2013;4(4):177‐191. 23863200 10.4161/trns.25558 PMC3977918

[42] Chu HW , Cheng CW , Chou WC , et al. A novel estrogen receptor‐microRNA 190a‐PAR‐1‐pathway regulates breast cancer progression, a finding initially suggested by genome‐wide analysis of loci associated with lymph‐node metastasis. Hum Mol Genet. 2014;23(2):355‐367. 24009311 10.1093/hmg/ddt426

[43] Wang J , Yu XF , Ouyang N , et al. MicroRNA and mRNA interaction network regulates the malignant transformation of human bronchial epithelial cells induced by cigarette smoke. Front Oncol. 2019;9:1029. 31649886 10.3389/fonc.2019.01029 PMC6794608

[44] Chen S , Wang L , Yao X , et al. miR‐195‐5p is critical in REGgamma‐mediated regulation of wnt/beta‐catenin pathway in renal cell carcinoma. Oncotarget. 2017;8(38):63986‐64000. 28969047 10.18632/oncotarget.19256 PMC5609979

[45] Zhao DL , Wu QL . Effect of inhibition to Yes‐related proteins‐mediated Wnt/beta‐catenin signaling pathway through miR‐195‐5p on apoptosis of gastric cancer cells. Eur Rev Med Pharmacol Sci. 2019;23(15):6486‐6496. 31378888 10.26355/eurrev_201908_18532

[46] Dai J , Wei R , Zhang P , Kong B . Overexpression of microRNA‐195‐5p reduces cisplatin resistance and angiogenesis in ovarian cancer by inhibiting the PSAT1‐dependent GSK3beta/beta‐catenin signaling pathway. J Transl Med. 2019;17(1):190. 31171023 10.1186/s12967-019-1932-1 PMC6551881

[47] Zhou WY , Zhang MM , Liu C , Kang Y , Wang JO , Yang XH . Long noncoding RNA LINC00473 drives the progression of pancreatic cancer via upregulating programmed death‐ligand 1 by sponging microRNA‐195‐5p. J Cell Physiol. 2019;234(12):23176‐23189. 31206665 10.1002/jcp.28884

[48] Chai L , Kang XJ , Sun ZZ , et al. MiR‐497‐5p, miR‐195‐5p and miR‐455‐3p function as tumor suppressors by targeting hTERT in melanoma A375 cells. Cancer Manag Res. 2018;10:989‐1003. 29760567 10.2147/CMAR.S163335 PMC5937487

[49] Luo Q , Zhang Z , Dai Z , et al. Tumor‐suppressive microRNA‐195‐5p regulates cell growth and inhibits cell cycle by targeting cyclin dependent kinase 8 in colon cancer. Am J Transl Res. 2016;8(5):2088‐2096. 27347317 PMC4891422

[50] Bai J , Xu J , Zhao J , Zhang R . lncRNA SNHG1 cooperated with miR‐497/miR‐195‐5p to modify epithelial‐mesenchymal transition underlying colorectal cancer exacerbation. J Cell Physiol. 2020;235(2):1453‐1468. 31276207 10.1002/jcp.29065

[51] Zheng J , Xu T , Chen F , Zhang Y . MiRNA‐195‐5p functions as a tumor suppressor and a predictive of poor prognosis in non‐small cell lung cancer by directly targeting CIAPIN1. Pathol Oncol Res. 2019;25(3):1181‐1190. 30637589 10.1007/s12253-018-0552-z PMC6614139

[52] Chen S , Chen X , Xiu YL , Sun KX , Zhao Y . MicroRNA‐490‐3P targets CDK1 and inhibits ovarian epithelial carcinoma tumorigenesis and progression. Cancer Lett. 2015;362(1):122‐130. 25819031 10.1016/j.canlet.2015.03.029

[53] Shen J , Xiao Z , Wu WK , et al. Epigenetic silencing of miR‐490‐3p reactivates the chromatin remodeler SMARCD1 to promote Helicobacter pylori‐induced gastric carcinogenesis. Cancer Res. 2015;75(4):754‐765. 25503559 10.1158/0008-5472.CAN-14-1301

[54] Xu X , Chen R , Li Z , et al. MicroRNA‐490‐3p inhibits colorectal cancer metastasis by targeting TGFbetaR1. BMC Cancer. 2015;15:1023. 26714817 10.1186/s12885-015-2032-0 PMC4696296

[55] Zhang F , Wu A , Wang Y , Liu J . miR‐490‐3p functions as a tumor suppressor in glioma by inhibiting high‐mobility group AT‐hook 2 expression. Exp Ther Med. 2019;18(1):664‐670. 31258704 10.3892/etm.2019.7606 PMC6566118

[56] Fan H , Zhang YS . miR‐490‐3p modulates the progression of prostate cancer through regulating histone deacetylase 2. Eur Rev Med Pharmacol Sci. 2019;23(2):539‐546. 30720161 10.26355/eurrev_201901_16866

[57] Kang NN , Ge SL , Zhang RQ , Huang YL , Liu SD , Wu KM . MiR‐490‐3p inhibited the proliferation and metastasis of esophageal squamous cell carcinoma by targeting HMGA2. Eur Rev Med Pharmacol Sci. 2018;22(23):8298‐8305. 30556870 10.26355/eurrev_201812_16527

[58] Ou Y , He J , Liu Y . MiR‐490‐3p inhibits autophagy via targeting ATG7 in hepatocellular carcinoma. IUBMB Life. 2018;70(6):468‐478. 29676845 10.1002/iub.1715

