# Supplementary figures and images for: Exostosin1 as a novel prognostic and predictive biomarker for squamous cell lung carcinoma: A study based on bioinformatics analysis
Source: Cancer Med. 2020 Dec 13;10(8):2787–801. doi: 10.1002/cam4.3643 (PMC8026939; doi:10.1002/cam4.3643)

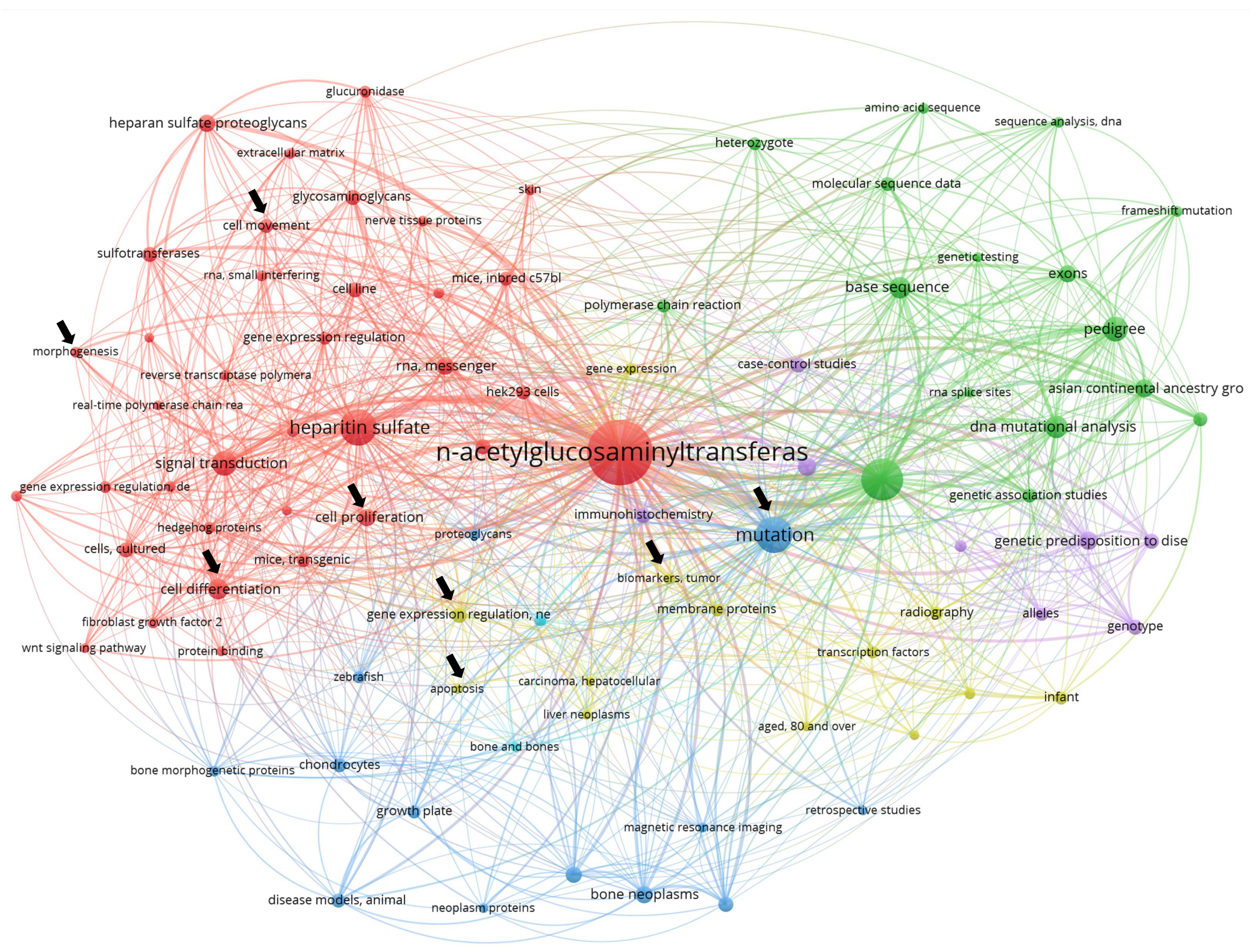

Supplement: Supplementary file 1 — Fig S1 [file CAM4-10-2787-s001.tif]
